# The Haiti cardiovascular disease cohort: study protocol for a population-based longitudinal cohort

**DOI:** 10.1186/s12889-020-09734-x

**Published:** 2020-11-01

**Authors:** Jean Lookens, Olga Tymejczyk, Vanessa Rouzier, Caleigh Smith, Fabyola Preval, Inddy Joseph, Raynold Jean Baptiste, Joscar Victor, Patrice Severe, Sandra Apollon, Emelyne Dumont, Guyrlaine Forestal, Stephano St. Preux, Vanessa Rivera, Grace Seo, Benedict Charles, Wilson Ariste, Justin Kingery, Jessy Devieux, Serena Koenig, Denis Nash, Daniel Fitzgerald, Monika Safford, Marie Marcelle Deschamps, Jean Pape, Margaret McNairy

**Affiliations:** 1 Haitian Group for the Study of Kaposi’s Sarcoma and Opportunistic Infections (GHESKIO), Port-au-Prince, Haiti; 2 CUNY Institute for Implementation Science in Population Health, New York, NY USA; 3 Center for Global Health, Weill Cornell Medicine, New York, NY USA; 4 Division of General Internal Medicine, Weill Cornell Medicine, New York, NY USA; 5 Department of Health Promotion and Disease Prevention, Stempel College of Public Health and Social Work, Miami, FL USA; 6 Division of Global Health Equity, Brigham and Women’s Hospital, Boston, MA USA

**Keywords:** Cardiovascular medicine, Hypertension, Epidemiology, Public health

## Abstract

**Background:**

Cardiovascular disease (CVD) is the leading cause of mortality among Haitians, having surpassed HIV in the last decade. Understanding the natural history of CVD in Haitians, including the age of onset, prevalence, incidence, and role of major risk factors and social determinants, is urgently needed to develop prevention and treatment interventions.

Aim 1: Establish a population-based cohort of 3000 adults from Port-au-Prince and assess the prevalence of CVD risk factors and diseases and their association with social and environmental determinants.

Aim 2: Determine the incidence of CVD risk factors and CVD during 2–3.5 years of follow-up and their association with social and environmental determinants.

**Methods:**

The Haiti CVD Cohort is a longitudinal observational study of 3000 adults > 18 years in Port-au-Prince (PAP), Haiti. The study population is recruited using multistage random sampling from census blocks. Adults receive blood pressure (BP) measurements in the community and those with elevated BP are referred to the Groupe Haitien d’Etude Sarcome de Kaposi et des Infections Opportunistes Clinic for care. After informed consent, participants undergo a clinical exam with medical history. BP, electrocardiogram, echocardiogram, a study questionnaire on health behaviors, and laboratory specimens. Every 6 months, BP is remeasured. At 12 and 24 months, clinical exams and questionnaires are repeated. Labs are repeated at 24 months. Adjudicated study outcomes include the prevalence and incidence of CVD risk factors (hypertension, diabetes, obesity, dyslipidemia, kidney disease, inflammation, poor diet, smoking, and physical inactivity) and events (myocardial infarction, heart failure, stroke, and CVD mortality). We also measure social determinants including poverty. Depression, stress, social isolation, food insecurity, and lead exposure. Blood, urine, and stool samples are biobanked at study enrollment.

**Discussion:**

The Haiti CVD Cohort is the largest population-based cohort study evaluating CVD risk factors and CVD among adults in urban Haiti with the goal of understanding the drivers of the CVD epidemic in Haiti. Study outcomes are comparable with existing international cohorts, and the biobank will provide important data for future research. Our goal is to translate findings from this study into pragmatic prevention and treatment interventions to fight the CVD epidemic in Haiti.

## Background

Cardiovascular disease (CVD) is now the leading cause of adult mortality in Haiti, having surpassed HIV in the last decade [1–4]. In 2017, the World Health Organization (WHO) reported that CVD is “the number one cause of death globally” [5]. This emerging CVD epidemic in resource-poor settings is likely due to an increase in traditional CVD risk factors, such as smoking, hypertension (HTN), and diabetes, as well as other social and environmental determinants such as poverty, stress, social isolation, depression, the effect of food insecurity on dietary habits, and environmental lead exposure [6–10]. With CVD being the largest driver of global mortality, the WHO has ambitiously targeted a one-third reduction in premature deaths from CVD and other non-communicable diseases by 2025 [11, 12]. The first step towards achieving this goal in Haiti is to understand the epidemiology, natural history, and drivers of CVD in this setting.

Existing data on the CVD epidemic in Haiti is primarily from modeling studies and cross-sectional clinic-based cohorts. Modeling from the Global Burden of Disease Project estimated that one-third of deaths in Haiti across all ages were due to CVD in 2016, in comparison to HIV which was estimated to cause 5.6% deaths [4]. In a systematic review of 18 countries in the Caribbean and Latin American regions, Haiti had the highest stroke mortality rate, more than double that of its neighbor, the Dominican Republic [13]. Moreover, the median age of stroke in Haiti is approximately 10 years lower than the average age of stroke in other developing countries, with a median age of 61 years for ischemic stroke and 52 years for hemorrhagic stroke in Haiti [14]. More recently, Haiti’s 2017 Demographic Health Survey (DHS) revealed that HTN was the most common CVD risk factor in Haiti with a prevalence of 49% in women and 38% in men ages 35 to 64 [15]. Our data from a smaller community-based survey of slum residents in Port-au-Prince (PAP) in 2016 found the prevalence of HTN among young Haitians was higher than similar aged Blacks in the US from historical cohort studies [16, 17].

The objective of the Haiti CVD Cohort study is to establish the first longitudinal cohort of Haitian adults to measure the prevalence and incidence of CVD risk factors and events and examine the role of associated social determinants. CVD risk factors include HTN, diabetes, renal disease, obesity, smoking, poor diet, physical inactivity, and inflammation. CVD events include myocardial infarction (MI), heart failure (HF), stroke, and CVD mortality. Our goal is to use these data to identify drivers of HTN and CVD events that can be targeted with prevention and treatment interventions to ultimately curb this epidemic.

### Formative research

In 2016, we conducted a small community-based study of HTN and other CVD risk factors in four slums in downtown PAP [17]. We found that 50% of the adult population was aged 18–30 years, 58% were women, and the age-standardized HTN prevalence, assessed via single-day measurement, was 28.5% [16]. In particular, we documented potentially high levels of early-onset HTN, with a HTN prevalence of 12% among young Haitian adults aged 18–30 years, in contrast to a prevalence of 3.4–5.3% among similarly-aged Black Americans in historical US cohorts [18–20]. Prevalence of other typical CVD risk factors were low (smoking 7%, diabetes 1%, and obesity 11%), suggesting that additional social and environmental determinants may contribute to the burden of HTN in PAP [7].

**Fig. 1:**
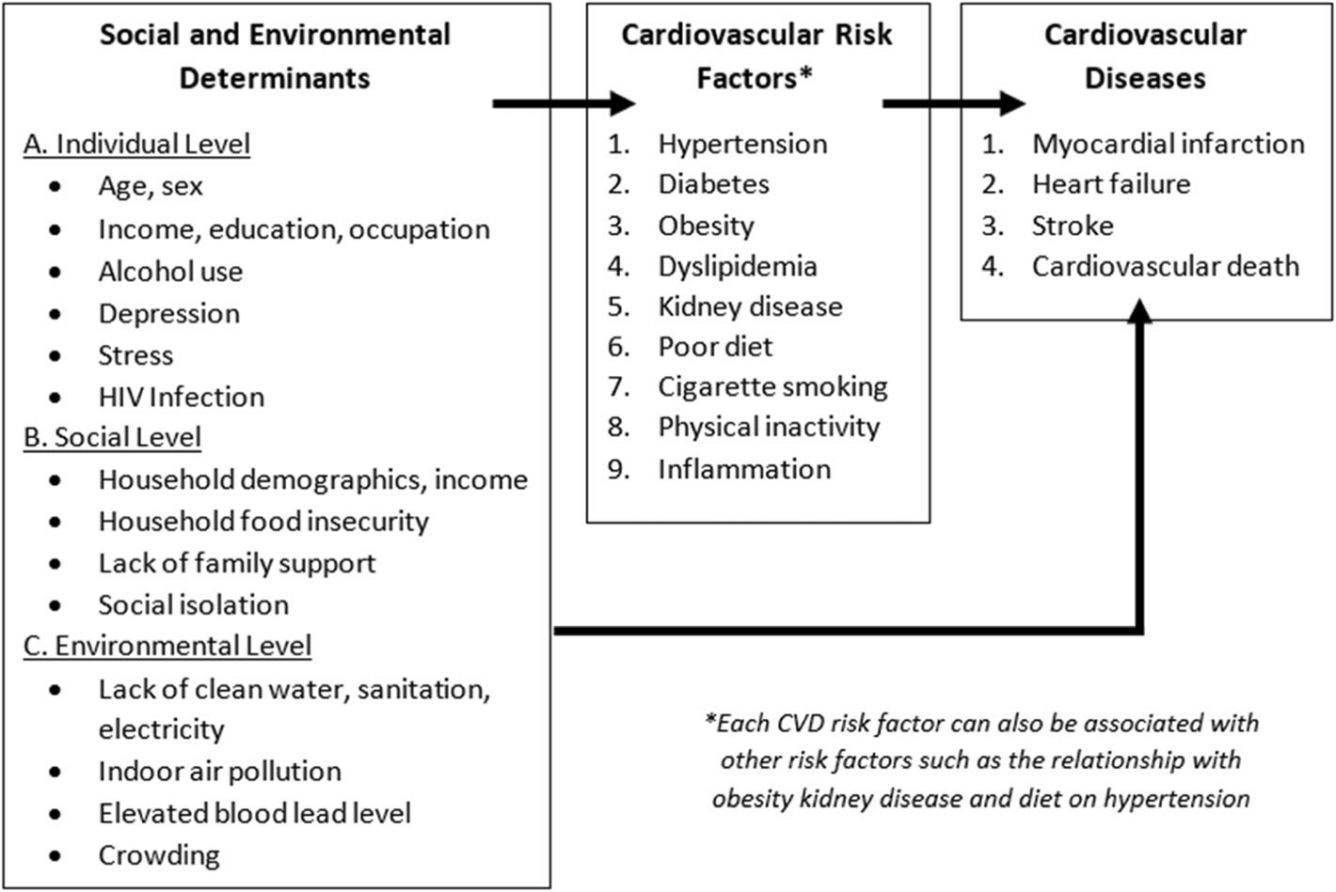
Adapted Social Ecological Model of CVD in Haiti

These findings and discussions with the Collège Haïtien de Cardiologie led us to hypothesize that other poverty-related social determinants may be contributing to HTN in Haiti. Figure 1 uses the social ecological theoretical model [6–10] to illustrate the potential relationship between social and environmental determinants, risk factors, and diseases in Haiti. Examples of social determinants that have been associated with CVD risk factors and events and are common in Haiti are poverty, depression, stress, social isolation, and educational attainment [21–27]. For example, depression has been independently associated with incident HTN [28–31] and also associated with a 4-fold increase in MI [32] and increased risk of cardiac death [33, 34]. Lead exposure is another poverty-related environmental determinant that even at low levels in the blood is associated with increased blood pressure (BP), CVD, and cardiac death [35–41]. Elevated blood lead levels in Haitians, specifically children, have also been reported, and local studies suggest that environmental lead exposure is widespread through soil, paint, contaminated water sources, and improperly discarded batteries [42].

## Methods

### Study design and site

**Fig. 2:**
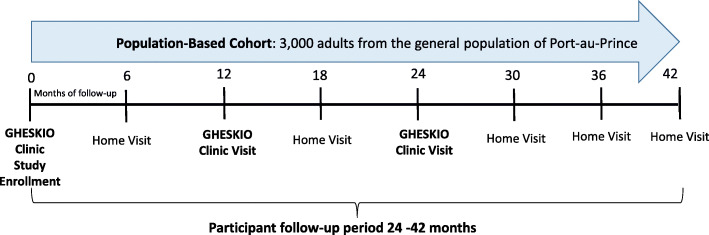
Initial Funding Period Timeline Overview

**Fig. 3:**
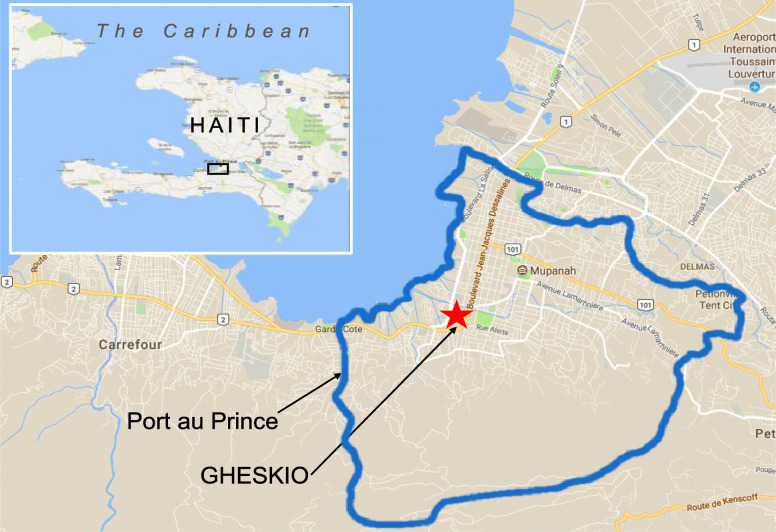
Map of Study Recruitment Area in Metropolitan Port au Prince and Research Site at GHESKIO

The Haiti CVD Cohort study is an observational longitudinal cohort study of Haitian adults who reside in metropolitan PAP. The initial funding period activities are shown in Fig. 2. The study site is the clinic established by Groupe Haitien d’Etude Sarcome de Kaposi et des Infections Opportunistes (GHESKIO), which is a large public health clinic in the center of downtown PAP (Fig. 3). GHESKIO was originally established in 1982 as an HIV clinic and has expanded to include other infectious and chronic diseases including CVD and HTN. Its primary catchment area includes metropolitan PAP with a focus on health care delivery in the adjacent slum neighborhoods.

#### Study population and sampling methodology

The study population includes 3000 adults of age > 18 years from metropolitan PAP. Potential participants are identified using multistage random sampling to identify potential participants [43]. The sampling frame is composed of census blocks of approximately 100–200 buildings, previously enumerated by the Institut Haitien de Statistique et d’Informatique (IHSI) [44–46]. Blocks experiencing political violence at the time of sample design in November 2018 or entirely commercial areas were excluded. Approximately 2045 waypoints across census blocks were then randomly assigned using Geographic Information Software, with the number of waypoints per block proportional to its estimated population. These waypoints are identified with GPS devices by community health workers (CHWs) during recruitment activities.

Prior to study launch, CHWs were trained to achieve similar competency on study procedures, community sensitization, and BP measurement. During community recruitment, CHWs use standard study procedures to select the closest residential building to each waypoint within a 50-m radius. CHWs obtain verbal consent from a household resident > 18 years to conduct a household survey and generate a roster of persons currently living in the household, defined as having slept in the household at least once in the past 2 weeks. From the roster, up to 2 adults are randomly selected and invited for study participation. CHWs also measure BP on all household adults present. CHWs revisit selected households at least three times and make a minimum of three phone calls to complete the roster and contact the randomly selected adults for participation. All household members, regardless of study participation, are invited for repeated BP screening and clinical care at the GHESKIO clinic.

Study eligibility criteria include age ≥ 18 years; primary residence in the study area of PAP (with no plans to move in the next 24 months); ability to provide consent for study procedures; documented consent to undergo study procedures; and willingness to be contacted at a new residence if a move occurs. Exclusion criteria include serious medical conditions or cognitive impairment preventing study participation as judged by research physicians; or inability to speak and understand French or Creole. Potential study participants are accompanied to the GHESKIO clinic, where research staff provide an overview of the study, complete an eligibility checklist, and obtain written informed consent.

Prior work indicated 50% of the adult population was 18–30 years and 60% were women [16]. The plan was to assess sample composition at 6 months and this assessment revealed the age distribution was as planned but we were recruiting too many women. Therefore, after this time point, we oversampled men to achieve the target 40% male sample composition (see Appendix for details).

##### Study enrollment

Consenting participants complete study enrollment procedures at the GHESKIO clinic within 3 months of recruitment. Research staff collect detailed locator information, administer a baseline questionnaire, measure BP, height, weight and waist circumference, and assess for CVD symptoms and history. BP is measured using WHO and AHA guidelines [47–49], which include having the participant seated in a quiet place for 5 min with both feet on the ground and the arm supported at the heart level. BP is measured electronically using an automated sphygmomanometer (Omron HEM 907) that takes three measurements separated by 30 s while the participant is unobserved by research staff. Then, a research nurse and physician administer a survey about individual, social and environmental characteristics and health behaviors including validated instruments of mental health, social support, and food insecurity (see Table 2). They also perform a physical exam and electrocardiogram (ECG). All participants with HTN or a history of CVD have an echocardiogram (ECHO) performed. In addition, the first 300 participants without evidence of CVD will have an ECHO to assess for subclinical disease. To the extent possible, all laboratory specimens (blood, urine, and stool sample) are collected after fasting. Participants receive a meal and transportation reimbursement for each research visit.

##### Follow-up study visits

Study visits at 12 and 24 months occur at the GHESKIO CVD clinic and include a follow-up questionnaire, weight and BP measurement, and screening for CVD symptoms and potential CVD events. Repeat laboratory measures, collection of specimens for biobanking, and an ECG are done at the 24-month study visit. A repeat ECHO is performed at 24 months on participants who received one at study enrollment.

In addition to clinic visits, CHWs perform home visits at 6 and 18 months for all participants, and every 6 months after 24 months for participants with longer follow-up, to update locator information, measure BP, and ask about CVD symptoms and potential CVD events. To encourage retention and minimize missing data, CHWs call study participants every 3 months to confirm locator information. If needed, research staff conduct follow-up questionnaires by telephone with participants who move outside of PAP.

### Study outcomes and measures

The primary study outcomes are the prevalence of CVD risk factors and established CVD at baseline and the development of CVD risk factors and CVD events over follow-up, defined as shown in Table 1. The main exposures are social and environmental determinants including poverty, stress, social isolation, depression, food insecurity, and lead exposure, defined as shown in Table 2.

**Table 1:** Cardiovascular risk factor and disease definitions

Cardiovascular Risk Factors	Definition
Hypertension (HTN)	Average measurements from study enrollment of SBP > 140 mmHg or DBP > 90 mmHg; or at least 1 enrollment BP measurement of SBP > 160 mmHg or DBP > 100 mmHg; or self-reported antihypertensive treatment. Incident HTN is defined as any subsequent study visit BP measurement of SBP > 140 mmHg or DP > 90 mmHg, or initiation or continuation of antihypertensive medications. Stage I HTN is defined as SBP 140–159 mmHg or DBP 90–99 mmHg; Stage 2 HTN is defined as SBP > 160 mmHg or DBP > 100 mmHg.
Diabetes	Fasting glucose > 126 mg/dL or non-fasting glucose > 200 mg/dL, or taking diabetes medications [68, 69]
Obesity	Body mass index > 30 kg/m^2^
Dyslipidemia	ACC/AHA guidelines for LDL levels based on risk category [70]
Kidney Disease	Urine albumin to creatinine ratio > 30 mg/g or eGFR < 60 mL/min/1.73 m^2^ as calculated using the CKD-Epi equation [71–74]
Poor diet	< 1 serving of fruits or vegetables per day, added salt to food, or > 1 fried food per day per the WHO STEPS survey definition [48, 75]
Smoking	Self-reported current tobacco smoking
Physical inactivity	< 150 min of moderate activity, or < 75 min of vigorous activity, or < 600 MET minutes per week [76]
Inflammation	hsCRP > 3 mg/L [77, 78]
**Cardiovascular Events**^**a**^	
Myocardial Infarction (MI)	Universal Definition of MI [79] and an AHA position statement [80] based on three major domains: clinical signs and symptoms consistent with ischemia, a rising and/or falling pattern of cardiac biomarkers over at least 6 h, and ECG or echocardiographic findings consistent with ischemia based on the Minnesota Criteria [81]
Heart Failure (HF)	Defined by clinical signs and symptoms based on the Framingham clinical diagnosis of HF [82], and adjudication is based on clinical symptoms, imaging findings, and b-type natriuretic peptide levels [82, 83]. We will classify the type of HF as HF with reduced ejection fraction (EF) or preserved EF based on echocardiographic readings.
Stroke	Based on consensus guidelines and defined as a clinical syndrome consisting of rapidly developing clinical signs of focal (or global in case of coma) disturbance of cerebral function lasting > 24 h or leading to death with no apparent cause other than a vascular origin; imaging findings consistent with infarction may be used even if symptoms resolve in < 24 h [84].
Cardiovascular Disease Death	Following recommendations set forth in Luepker, et al., using all available information including an interview with next of kin; CVD death includes death due to MI, HF, or stroke [80].

^a^ All events will be adjudicated by committee

**Table 2:** Study measures by study visit

Study Measures	Study Visit				
	0 M	6 M	12 M	6 M	24 M
**Individual Characteristics**					
**Locator information**	X		X		X
**Sociodemographics**: age, sex	X				
**Socioeconomic factors:** income, education, occupation, marital status, children	X				
**Medical History:** history of CVD risk factors, CVD, HIV, tuberculosis	X				
**Family History:** stroke or MI in parents and 4 oldest siblings	X				
**Health status:** Short Form questionnaire [50, 51]	X				
**Medications:** complete list taken in the last 2 weeks prescription, non-prescription, and herbal or traditional medicine	X		X		X
**Health behaviors:** current tobacco **smoking** and number of pack years [48], **alcohol** (number of alcoholic beverages consumed per day and week using NIDA cutoffs by gender [52–55]), **physical activity** (WHO Global Physical Activity Questionnaire [56])	X		X		X
**Places lived**	X		X		X
**Diet:** WHO STEPS Diet Survey [48]	X		X		X
**Negative emotions:** Depression (PHQ-9), Stress (Perceived Stress Scale-4) [57, 58]	X				X
**Health access/utilization**: hospitalization and clinic visits	X		X		X
**Social Characteristics**					
**Household demographics and socioeconomic factors**: family size, dependents, family income, housing stability	X				
**Household Food Insecurity**: Short Form Household Food Security Scale [59]	X				X
**Social isolation:** Multidimensional Scale of Perceived Social Support [60]	X				X
**Neighborhood stress and violence**: Neighborhood Collective Efficacy [61, 62], City Stress Inventory [63, 64]	X				X
**Traditional medicine practices**	X				X
**Environmental Characteristics**					
**Water and Sanitation**: clean water, indoor plumbing, latrine	X				
**Electricity**	X				
**Indoor cooking and type of cooking**	X				
**Lead exposure history:** lead paint, cookware, occupation	X		X		X
**Crowding**: population density of neighborhood using IHSI census data [65]	X				
**Clinical Measures**					
**Physiologic measures**: height, weight, waist circumference, left arm circumference	X		X		X
**Blood pressure (BP):** every 6 months in clinic or community	X	X	X	X	X
**Electrocardiogram**	X				X
**Echocardiogram:** performed on participants with signs or symptoms of heart failure, MI, stroke, or hypertension and 300 participants without symptoms	X				X
**Biologic specimens**: fasting blood, urine (albuminuria, cholesterol, creatinine, CRP, glucose, hemoglobin, HIV, lead level, lipids, white blood cells)	X				X
**Biobanked specimens**: whole blood, plasma, serum, urine, stool sample at enrollment and only blood will be collected at 24 months.	X				X
**CVD symptoms and event screening**: WHO Angina Questionnaire [66, 67], Questionnaire to Verify Stroke-free Status [67], and self-reported CVD events	X		X		X
**Medical record abstraction:** diagnoses codes (ICD-9), laboratory measures, diagnostic imaging, and cause of death among participants who receive clinical care from the GHESKIO clinic, a GHESKIO-affiliated hospital, or other health facility					

CVD outcomes will be adjudicated by a team of experts following national and international guidelines [78–83]. Medical records will be retrieved for each potential event reported by the participant or proxy, and two adjudicators will review the case, with disagreements resolved by committee. Agreement between adjudicators will be tracked with retraining if kappa falls below 0.8.

### Power and sample size calculations

The sample size is 3000 participants. Based on our prior work, we assumed a 96% household response rate and 91% of households having at least 2 adults available for study participation [16, 17]. We anticipate needing to identify and recruit from approximately 1963 households across 2045 GPS points. The resulting sample of approximately 3750 adults invited to participate allows for an individual non-response rate of 20% to constitute a cohort of 3000 participants. If the non-response rate exceeds 20%, additional GPS points will be added according to randomization procedures to reach the total study population of 3000 participants.

Our power considerations focus on HTN as the most prevalent CVD risk factor. A power analysis was conducted to determine the minimum detectable odds ratios (OR) between a determinant and HTN in a sample of 3000 adults with 80% power and two-tail alpha of 0.01 to adjust for multiple comparisons. For example, assuming 20% hypertension prevalence, we will be able to detect ORs ranging from 1.35 for food insecurity (estimated 50% prevalence) to 1.98 for elevated blood lead level > 5 μg/dL,(~ 5% prevalence) [85].

### Statistical methods

We will evaluate participant characteristics using summary statistics to identify outliers and data trends. Prevalence of CVD risk factors will be reported for categorical risk factors (e.g., HTN), and mean and standard deviation will be estimated for continuous risk factors (e.g., BP, lipid levels). We will also estimate the prevalence of baseline CVD including a history of MI, HF, and stroke. We will weight prevalence estimates to the general population distribution of PAP using 2015 Haitian census data [65], incorporating sampling weights for age and sex in our survey design. Prevalence estimates will also be stratified by gender and age groups to be comparable to existing US datasets and published estimates.

We will use multivariable logistic regression to assess the association between social determinants and CVD risk factors and history of CVD, incorporating sampling weights. We will assess the association between social determinants that are common in Haiti (stress, social isolation, depression, food insecurity, and blood lead level) and HTN. We will also examine the association of determinants with other CVD risk factors and diseases. We will assess for interaction between determinants and age and sex on CVD risk factors and diseases. For outcomes that are common, such as HTN, we will consider the use of binomial regression with Poisson distribution [86]. Linear and Poisson models will be used to evaluate associations between social determinants and continuous outcomes (BP) and count data (presence of CVD), respectively.

We will calculate the incidence rate of each CVD risk factor per 1000 person years (PY) of follow-up among participants without the respective CVD risk factor at baseline using a Horvitz-Thompson type estimator accounting for unequal sampling weights [87]. Similarly, we will calculate the incidence rate of each CVD outcome among participants without a history of CVD at baseline. Follow-up time for incidence rate calculations will be measured from the date of the participant’s study enrollment to the onset of a CVD risk factor or the date of the CVD event, the participant’s censor date (loss-to-follow up or study end), or death, whichever happens first. We will also calculate and plot the cumulative probability of onset of new CVD risk factors over time using the cumulative incidence function, accounting for competing risks and/or informative censoring for clinical outcomes such as death.

We will use hazard ratios (HR), with Cox proportional hazards regression accounting for interval censored data, to examine the associations between social determinants, CVD risk factors, and CVD outcomes [88, 89]. We will use Fine and Gray model to estimate competing risk of incident CVD risk factors or diseases with the competing risk of death [90]. We will perform sensitivity analyses using survey weight adjusted Cox regression of right-censored data and Kovalchick and Pfeiffer methods for competing risk for survey data [90]. For longitudinal data (e.g. repeated BPs as a continuous measure), we will use statistical methods suited for longitudinal data analysis (GEE or linear mixed models with appropriate weights) [91]. For social determinants found to be significantly associated with a CVD risk factor or disease, adjusted associations from models will also be used to clarify the relative contribution of various determinants. We will do this by estimating the attributable fraction for key variables, which is based on both adjusted measures of association and proportion exposed [92].

## Discussion

To achieve the WHO’s 2025 CVD reduction target, we must better understand the epidemiology and natural history of CVD in resource-poor settings such as Haiti. This study provides a population-based cohort not selected on disease or exposure status to describe the natural history and drivers of the CVD epidemic in Haiti, with the goal to identify both prevention and treatment interventions.

The study’s recruitment strategy is based on CHWs, which are a centerpiece of GHESKIO’s work. GHESKIO has worked with CHWs for community-based HIV testing, TB screening, cholera vaccination, and community health [93–95]. CHWs are often from the same neighborhoods they serve which helps establish rapport and trust with households. Thus far, with 1480 participants recruited, CHWs have been able to correctly identify all GPS points and have a 95% household participation acceptance rate. Additionally, CHWs provide free BP screening for all household members and refer them to the GHESKIO clinic for additional counseling and care, regardless if the individual was selected as a potential participant. The study provides an opportunity to explore the acceptability of community-based BP screening using CHWs for future prevention and treatment interventions. Additionally, our household rosters will provide valuable data on the household structure in the dynamic setting of metropolitan PAP.

This study has vastly increased GHESKIO’s CVD research and clinical capacity through training and deepening collaborations with local stakeholders on chronic disease. For example, we have trained three internal medicine physicians on bedside cardiac echocardiography. We have developed guidelines for the diagnosis and treatment of HTN in primary care customized to Haiti [96] and we have expanded our partnership with the Collège Haïtien de Cardiologie to explore ongoing research questions and priorities for future research. Together we held the first national conference on HTN in Haiti with international experts and developed working guidelines for first-line HTN treatment at GHESKIO. Expanding resource collaborations to include chronic diseases in resource-limited countries like Haiti is essential in responding to the alarming increase in the CVD epidemic in these settings.

There are both strengths and challenges to our recruitment strategy. Based on our prior work, community-based recruitment in urban PAP from the household level is both feasible and highly acceptable. It also provides an opportunity for public health education about HTN and free screening and counseling. Challenges include lack of resources for mobile clinics, which would allow more convenient community-based enrollment. Instead, travel to the GHESKIO clinic is required for enrollment, which may be inconvenient to healthy participants who work during the day, particularly younger and male individuals. We have added weekend enrollment and a second GHESKIO site to improve accessibility. Unexpectedly, recruitment has been interrupted several times due to political instability in Haiti; the most recent standstill lasted over 3 months and shut down all public transport, making it impossible for participants and staff to travel to GHESKIO and around the city.

Strengths of this study include random selection of geographic locations and participants, which increases the generalizability of study findings to other residents living in these communities. Secondly, we use standardized measurement protocols for BP and CVD event definitions from the WHO and AHA, which will facilitate comparisons across settings. Further, we have included several household-level determinants such as neighborhood violence and cohesiveness, and social and family support, which may provide insight on future interventions that are community- or family-based rather than solely individual-based. Another strength of the study is our biobank of laboratory specimens which will be valuable for future research on genetics, metabolomics, and the microbiome to better understand the pathogenesis of CVD.

This study is an urban cohort and similar studies in rural areas are needed. Other challenges may be difficulty in recruiting men, limited medical records, and lack of a vital registry in Haiti to collect hospitalization, vital status and cause of death data. Additionally, we anticipate we may have limited CVD incident outcomes given the young age of our cohort and limited follow-up time for the initial funding period. Ideally, this is the first step in establishing a longitudinal cohort that can expand in size and duration over time.

### Study status

The study began enrollment on March 7, 2019 and enrollment is ongoing. Follow-up activities are currently being completed for enrolled participants.

## Conclusion

The Haiti CVD Cohort study establishes a population-based longitudinal cohort of adults in Port-au-Prince, Haiti, and responds to the urgent need of measuring and understanding the CVD epidemic and its drivers in resource-poor settings. With a robust set of Haiti-adapted, US-comparable study measures including clinical, laboratory, social, environmental, individual, and household determinants, the Haiti CVD Cohort study will provide much needed insight into the epidemiology and natural history of CVD in Haiti and will build a platform for comparisons with existing US-based and other global CVD cohorts. This initial assessment will provide urgently needed CVD risk factor and CVD burden estimates that will inform national and international CVD prevention, diagnosis, and treatment guidelines.

## Data Availability

The datasets used and/or analyzed during the current study are available from the corresponding author on reasonable request. Data request should be submitted to Dr. Margaret McNairy (mam9365@med.cornell.edu) who will review the data request with Haiti GHESKIO Site PI, Dr. Jean Pape and the study’s Observational Monitoring Board for approval.

## Appendix

### Revised sampling frame

Per the study protocol, the sex distribution of the sample is assessed every 6 months to determine if the proportion of women is higher than 60%. After 6 months of enrollment, 73% of our study population were women. Therefore, we amended the recruitment protocol in order to increase the proportion of men to 40%, a projection based on the household composition and participation rates to date. The proposed recruitment and random selection changes were as follows:

1. If a household has 1–2 adults, no change to the protocol is needed, as there is no need for randomization. All adults will still be selected.
2. If a household has 3 or more adults:
  - If there are no men, randomly select two people as usual.
  - If there are men, randomly select only 1 woman per household and select all men in the household.

## Abbreviations

CVD: Cardiovascular disease
PAP: Port-au-Prince
BP: Blood pressure
GHESKIO: Groupe Haitien d’Etude Sarcome de Kaposi et des Infections Opportunistes
WHO: World Health Organization
HTN: Hypertension
DHS: Demographic and Health Survey
MI: Myocardial infarction
HF: Heart failure
IHSI: Institut Haitien de Statistique et d’Informatique
CHW: Community health workers
ECG: Electrocardiogram
ECHO: Echocardiogram
OR: Odds ratio
